# Infectious Disease Spreading Fought by Multiple Vaccines Having a Prescribed Time Effect

**DOI:** 10.1007/s10441-022-09452-4

**Published:** 2022-11-15

**Authors:** Rinaldo M. Colombo, Mauro Garavello

**Affiliations:** 1 INdAM Unit & Department of Information Engineering, University of Brescia, Brescia, Italy; 2 Department of Mathematics and Its Applications, University of Milano - Bicocca, Milan, Italy

**Keywords:** Vaccination strategies, Macroscopic modeling of disease propagation, PDEs in epidemiology

## Abstract

We propose a framework for the description of the effects of vaccinations on the spreading of an epidemic disease. Different vaccines can be dosed, each providing different immunization times and immunization levels. Differences due to individuals’ ages are accounted for through the introduction of either a continuous age structure or a discrete set of age classes. Extensions to gender differences or to distinguish fragile individuals can also be considered. Within this setting, vaccination strategies can be simulated, tested and compared, as is explicitly described through numerical integrations.

## Introduction

We propose a modeling framework to simulate the global process of a vaccination campaign to fight the spreading of an epidemic. Vaccines, possibly with different characteristics, are dosed to susceptible individuals. Each vaccine is identified by the efficiency and the duration of the protection it provides. In our model also individuals that recovered from the disease are immunized for a prescribed time period, after which they get back to be susceptible. In the age structured version, these times and efficiencies are assumed to be age dependent.

A common strategy to insert vaccination and in particular the loss of immunization in a SIR type model consists in assigning to these phenomena a *rate*, typically proportional to the number of susceptible and vaccinated individuals. On the contrary, here we introduce the time at which individuals are vaccinated, account for the time dependent level of immunization provided by the vaccine and give a precise role to the duration of this immunization.

The proposed class of models relies on a deterministic and macroscopic description, developed on top of the SIR model, and displays an evolution which is inherently *“multiscale”*: a first time scale is that of the pathogen diffusion, which interacts at different time scales with the different vaccines and with the recovering from the disease. For a stochastic approach, we refer for instance also to Bertaglia et al. (2022), while a fuzzy approach is in Al-Qaness et al. (2020) and Regis et al. (2021). On the basis of the epidemic evolution described by the present model, consequences at the social or economic levels can be described as in Albi et al. (2021a) and Fabbri et al. (2021), for instance, or (Bernardi et al. 2022; Dimarco et al. 2020) where a kinetic model of wealth exchange is proposed. A summary of the historical development of macroscopic models for virus diffusion and vaccination is in Groppi and Della Marca (2018).

**Fig. 1:**
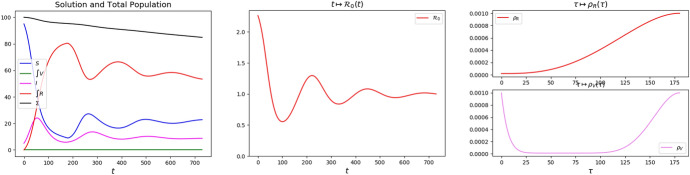
Solution to (2.4)–(2.9)–(2.10) with no vaccinations, i.e., $p\equiv 0$. Left, the graphs of $t \mapsto S (t)$, $t \mapsto \int _0^{T_V}V (t,\tau ){\textrm{d}{\tau }}$, $t \mapsto I (t)$, $t \mapsto \int _0^{T_R}R (t,\tau ){\textrm{d}{\tau }}$ and of their sum, labeled by $\Sigma$. Middle, the corresponding graph of $t \mapsto {\mathcal {R}}_0 (t)$, as defined in (2.8). Right, the graphs of the functions $\tau \mapsto \rho _R (\tau )$, above, and of $\tau \mapsto \rho _V (\tau )$, below, used in this and in the forthcoming integrations

**Fig. 2:**
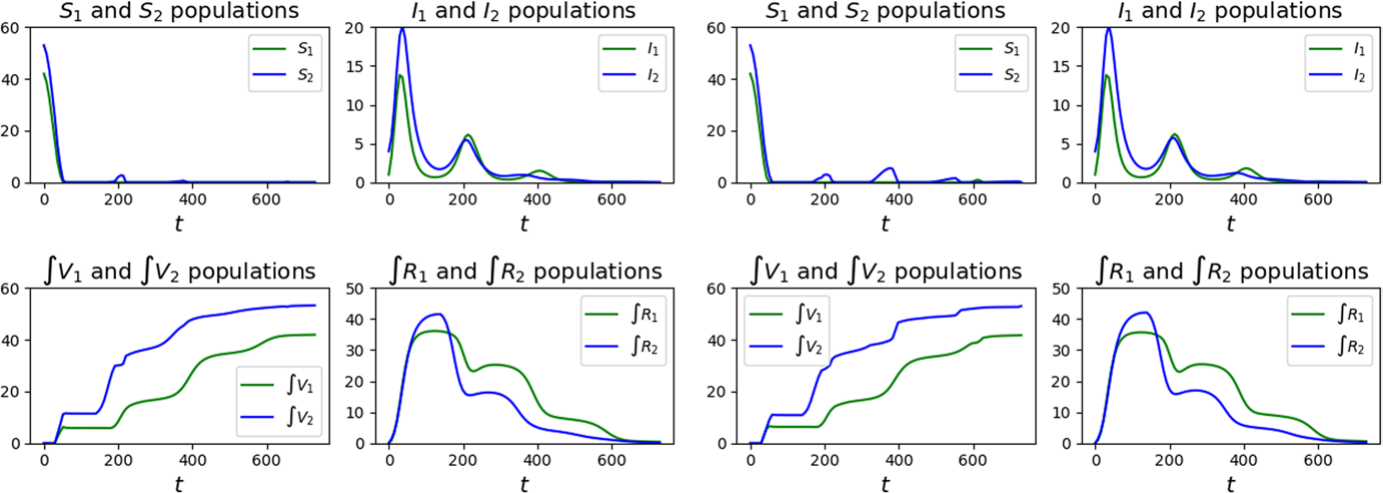
Graphs of the solutions to (4.3) with parameters (4.5)–(4.6), initial datum (4.7) and with the vaccination strategies Feedback, left, and Half–Half, right, as detailed in Sect. 4.1

**Fig. 3:**
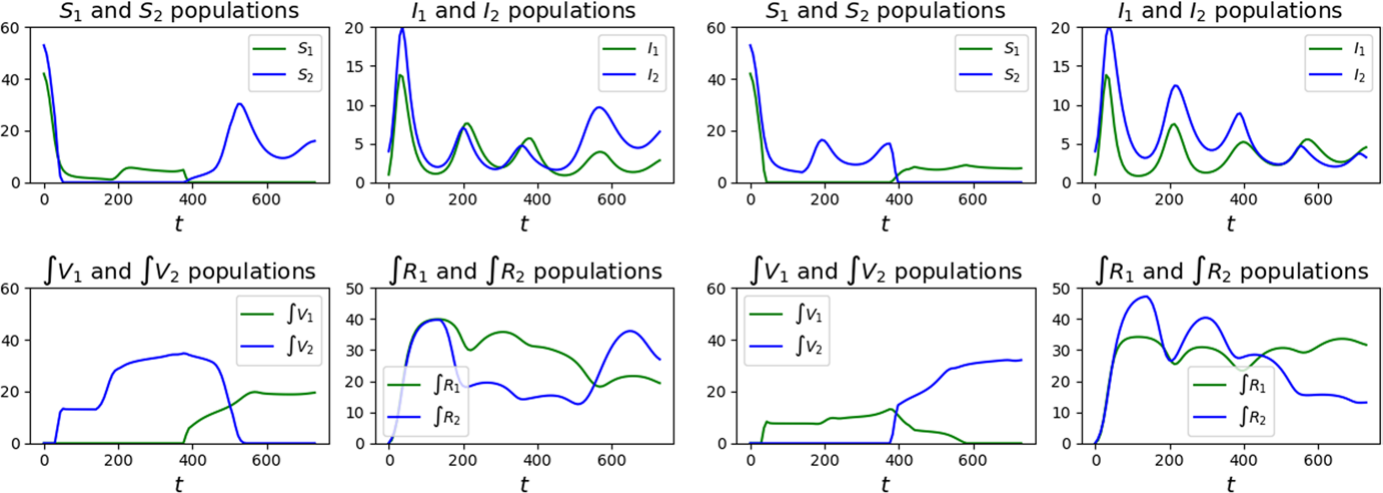
Graphs of the solutions to (4.3) with parameters (4.5)–(4.6), initial datum (4.7) and with the vaccination strategies Class 2 First, left, and Class 1 First, right, as detailed in Sect. 4.1

The interaction among the different populations, e.g., susceptible, infected, vaccinated and recovered, combined with the different time scales leads to the formation of oscillations or *epidemic waves* (Lemon and Mahmoud 2005). When no vaccination is dosed (e.g., Fig. 1), or even more when a very heavy vaccination campaign is in place (e.g., Fig. 2), then these waves fade out rather quickly. On the contrary, a relatively mild vaccination campaign hinders the virus propagation without stopping it, so that these epidemic waves become rather persistent (e.g., Fig. 3).

**Fig. 4:**
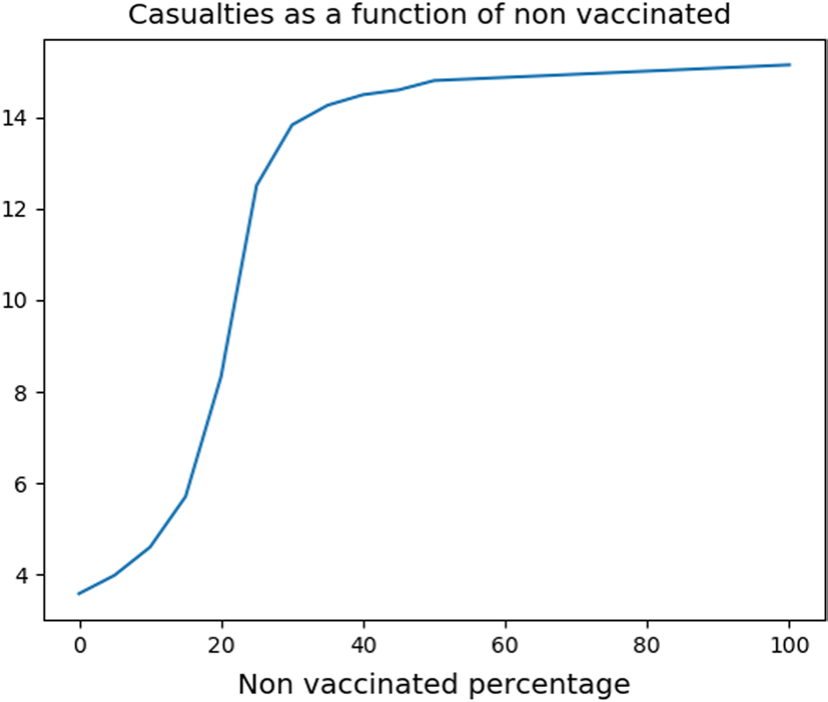
Casualties as a function of the non vaccinated percentage. Note the sharp increase starting already at a threshold of about $10\%$, somewhat justifying the term *“herd immunity”*. The corresponding values are in Table 1

The present model allows to test/compare different vaccination strategies. For instance, analyzing the number of casualties resulting from a vaccination campaign that leaves a fixed percentage, say $S_*$, of non vaccinated individuals shows a sort of *“herd immunity”* (Randolph and Barreiro 2020) effect. Indeed, the number of casualties suffers a sharp increment in correspondence to a threshold value $S_*$, roughly close to $10\%$ of the initial population (see Fig. 4).

The choice of the vaccination strategy gets even more relevant when different vaccines are available. It is realistic to imagine that different vaccines provide different levels of immunity for different time periods (Kai et al. 2021; Mukhopadhyay et al. 2021). Then, for instance, the use of a poor vaccine has a doubly negative effect. First, it does not ensure a good level of immunization and, second, may prevent vaccinated individuals to get a better vaccine as long as its effect is in place, see Sect. 3.1.

Age differences, too, require careful planning of vaccination campaigns. Consider for simplicity 2 classes: *“younger”* individuals are more infective, while *“older”* ones are more fragile. A vaccination strategy consisting in dosing exclusively the older ones first is not necessarily the best choice. Indeed, a campaign where the proportions of young and old dosed is carefully chosen according to the disease diffusion can reduce the number of casualties, even in the old class, see Sect. 4.1.

In the realizations of the present framework discussed below, we keep on purpose the number of populations to a minimum. It goes without saying that the extension to richer structures is easily achievable at the cost of only technical complications. The current literature provides various examples of multispecies/multicompartment models, often compared with real measurements, see for instance (Giordano et al. 2020; Parolini et al. 2021; Yang and Wang 2020).

We stress that the setting here introduced is amenable to consider, for instance, also movements in space, gender differences or the presence of more fragile individuals. These extensions, clearly, formally complicate the equations. However, their numerical treatment fits in the brief description in Appendix B and does not require the introduction of new or *ad hoc* algorithms. Movements in space can be comprised with a procedure similar to that used in Sect. 4 to introduce a continuous age structure, possibly introducing a further distinction among individuals having different destinations, see Colombo et al. (2020, 2022b) for further details. A different approach to diffusion in space is treated, for instance, in Pugliese and Milner (2018). The setting therein is based on stochastic ordinary differential equations, eventually leading to partial differential equations of second order in the space derivative (Pugliese and Milner 2018, Formula (13)). For a discussion of gender and age differences see (Russo et al. 2021, Table 1).

Aiming at a quantitative fitting with specific data reasonably requires to let the various functions and parameters defining the evolution (e.g., recovery rate, vaccine’s efficiency or duration, infectivity, $\ldots$) depend on time. The introduction of time dependencies may account, for instance, for seasonal effects, changes in lockdown policies, improvements in drug efficacy, $\ldots$ see Buonomo et al. (2019) and Merow and Urban (2020).

The next section is devoted to the simplest case of a single vaccine (whose effect has a prescribed duration) without age structure. Then, Sect. 3 deals with the concurrent use of multiple vaccines. Age structures, both continuous and discrete, are the subject of Sect.  4: here, in particular, the effects of vaccines depend on age. In Sect. 5 we address the issue of choosing proper values for the parameters and functions in the models we introduced, on the basis of Covid–19 related data, mostly related to the Italian situation, from the current literature.

## A Single Vaccine

We present here our framework in its simplest realization, namely considering a single vaccine and we test different vaccination strategies to control the spreading of the disease.

As a starting point (Groppi and Della Marca 2018, Formula (5)), consider the SIR model

2.1 $$\left\{ {\begin{array}{*{20}l} {\dot{S}} = - {\rho _{S} IS} \\ {\dot{I}} = {\rho _{S} IS - \theta I - \mu I} \\ {\dot{R}} = {\theta I} \\ \end{array} } \right.$$

where, as usual *S*, *I*, *R* are the number (or percentages) of Susceptible (*S*), Infected (*I*) and Recovered (*R*) individuals. The infectivity coefficient $\rho _S$, the recovery rate $\theta$ and the mortality rate $\mu$ are here considered to be constant; were they time dependent, only technical difficulties would arise. As is well known, in (2.1) the total number of individuals varies, actually diminishes, exclusively due to the mortality term, i.e., $\frac{{\textrm{d}{~}}}{{\textrm{d}{t}}} (S+I+R) = -\mu \, I$. When long time intervals are considered, it might be appropriate to include mortality also in the *S* and *R* equations, or also natality, typically only in the *S* equation. Other realizations might comprehend also time dependent immigration/emigration terms, for instance.

As a first step, we modify (2.1) to allow for recovered individuals to get re-infected, after a time $T_R$ from recovery. To this aim, we modify the unknown *R* to $R = R (t,\tau )$, the variable $\tau$ being the time since recovery, with $\tau \in [0, T_R]$:

2.2 $$\begin{aligned} \left\{ \begin{array}{l} \dot{S} = -\rho _S \, I \, S + R (t,T_R)\\ \dot{I} = \rho _S \, I \, S + \int _{{{0}^{T_{R}}}} \rho _R (\tau ) \, R (t,\tau ) {\textrm{d}{\tau }}\, I - \theta \, I - \mu \, I \\ \partial _t R + \partial _\tau R = - \rho _R \, R \, I\\ R (t,0) = \theta \, I \,. \end{array} \right. \end{aligned}$$

Here, the *R* compartment displays an *“internal dynamics”*, see Colombo et al. (2022b). In other words, $R (t,\tau )$ is the number of individuals at time *t* that recovered at time $t-\tau$. Elementary, though useful, is to note that the *R* in (2.2) and the variable bearing the same name in (2.1) have different dimensions. As above, the total number of individuals varies, namely diminishes, exclusively due to mortality, i.e.,

$$\begin{aligned} \frac{{\textrm{d}{~}}}{{\textrm{d}{t}}} \left( S (t) + I (t) +\int _0^{T_R} R (t,\tau ){\textrm{d}{\tau }}\right) = -\mu \, I (t) \,. \end{aligned}$$

The function $\rho _R = \rho _R (\tau )$ describes how easy/difficult it is that an *R* individual gets infected after time $\tau$ from recovery. A possible reasonable behavior of the map

2.3 $$\begin{array}{*{20}c} {\rho _{R} (\tau ) = \rho _{R}^{ - } + (\rho _{S} - \rho _{R}^{ - } )\;\Phi \left( {\frac{\tau }{{T_{R} }}} \right)} \\ {\Phi (s) = (4 - 3s)s^{3} } \\ \end{array}$$

**Fig. 5:**
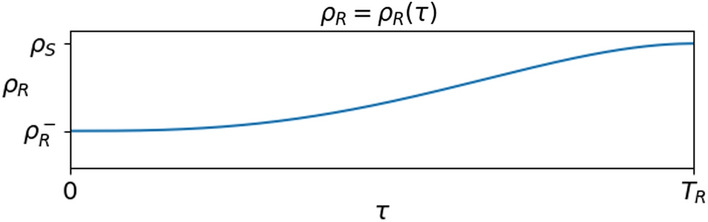
Left, a reasonable choice of the function $\rho _R$: at $\tau \approx 0$ we have $\rho _R (\tau ) \approx \rho _R^-$, a *small* ($\rho _R^- \ll \rho _S$) value quantifying the immunization resulting from recovering. As time from recovery passes, $\rho _R (\tau )$ increases and at time $T_R$ attains the value $\rho _S$ of susceptibles. Right, the actual expression used in the diagram on the left and in the numerical integrations in Sect. 2. The relevant properties of $\Phi$ are its continuity and monotonicity, from $\Phi (0) = 0$ to $\Phi (1)= 1$

$\tau \mapsto \rho _R (\tau )$ is depicted in Fig. 5. For $\tau$ near to 0, $\rho _R (\tau )$ equals $\rho _R^-$, a value far smaller than the infectivity coefficient $\rho _S$ in (2.1) or (2.2). As the time $\tau$ from recovery grows, also $\rho _R (\tau )$ grows and gets back to the value $\rho _S$ at time $T_R$, when recovered individuals return to be susceptible. The extension to $\rho _R$ depending also on *t* is immediate, as also that of letting $T_R \rightarrow +\infty$, as explicitly considered below.

In (2.2), the rate $\theta \, I$ at which infected individuals recover, tuned through the constant $\theta$, is the same as in (2.1). Each recovered individual after time $\tau = T_R$ from recovery gets back to being susceptible.

Remark that when considering a finite number of age classes $a_1, a_2, \ldots , a_k$ or a continuous age structure with $a \in {\mathbb {R}}_+$, then different ages may well have different times $T_R$, i.e., $T_R = T_R (a)$.

The effect of a vaccination that does not ensure permanent immunity is to some extent similar to the temporary immunization of recovered individuals as described above. A first difference is that immunization is obtained some time after being dosed. More relevant, vaccinations depend on a vaccination strategy, i.e., on the arbitrary choice of which and how many susceptibles are dosed at each time. Therefore, we introduce a new population, namely *V*, where $V (t,\tau )$ is the number of vaccinated individuals at time *t* that were dosed at time $t-\tau$, so that $\tau$ here is the time since vaccination.

We are thus lead to introduce the model

2.4 $$\begin{aligned} \left\{ \begin{array}{l@{}} \displaystyle \dot{S} = - \rho _S \, I \, S + V (t,T_V) + R (t, T_R) - p (t, S, V, I, R) \\ \displaystyle \partial _t V + \partial _\tau V = - \rho _V \, V \, I \quad \tau \in [0, T_V] \\ \displaystyle \dot{I} = \left( \rho _S \, S + \int _0^{T_V} \!\! \rho _V (\tau ) \, V (t,\tau ) {\textrm{d}{\tau }}+ \int _0^{T_R} \!\!\rho _R (\tau ) \, R (t,\tau ) {\textrm{d}{\tau }}- (\theta + \mu ) \right) I \qquad \\ \displaystyle \partial _t R + \partial _\tau R = - \rho _R \, R \, I\quad \tau \in [0, T_R] \\ \displaystyle V (t, 0) = p(t,S, V, I, R) \\ \displaystyle R (t, 0) = \theta \, I \,, \end{array} \right. \end{aligned}$$

where $T_V$ is the time when the immunization provided by vaccination terminates. Similarly to what is described above with reference to the function $\rho _R = \rho _R (\tau )$, now the function $\rho _V = \rho _V (\tau )$ describes how easy/difficult it is for an individual dosed at time $t-\tau$ to get infected at time *t*, i.e. after time $\tau$ from vaccination, for $\tau \in [0, T_V]$.

2.5 $$\begin{array}{*{20}c} {\rho _{V} (\tau ) = \rho _{V}^{ - } + (\rho _{S} - \rho _{V}^{ - } )\;\Psi \left( {\frac{\tau }{{T_{V} }}} \right)} \\ {\Psi (s) = \left( {1 - \frac{{27}}{4}s(1 - s)^{2} } \right)^{4} } \\ \end{array}$$

**Fig. 6:**
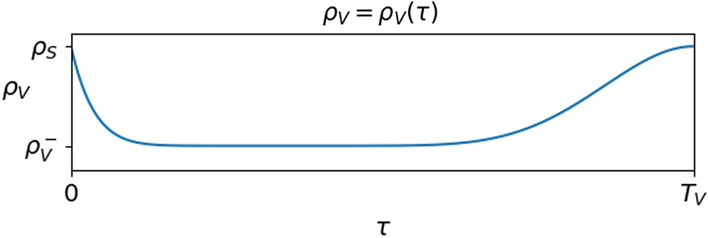
Left, a reasonable choice of the function $\rho _V$: at $\tau \approx 0$ we have $\rho _V (\tau ) \approx \rho _S$, since immunization is not immediate after being dosed. As time from vaccination passes, $\rho _V (\tau )$ decreases, reaches a lowest level $\rho _V^-$ and at time $T_V$ is back at the value $\rho _S$ of susceptibles. Right, the actual expression used in the diagram on the left and in the numerical integrations in Section 2. The relevant properties of $\Psi$ are its continuity, the fact that $\Psi (0) = \Psi (1)= 1$ and the kind of *plateau* near its minimum

**Fig. 7:**
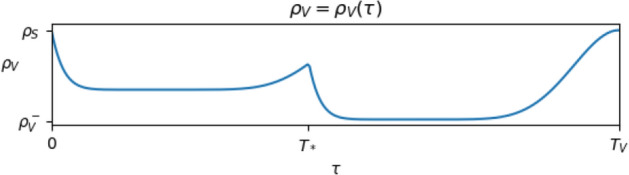
Qualitative behavior of a possible map $\tau \mapsto \rho _V (\tau )$ in the case of a vaccination consisting of 2 doses, whose effect ceases at time $T_V$. The second shot takes place at time $T_*$ after the first one. After time $T_V$ from the first dose, the protection provided by the vaccine expires, since $\rho _V (T_V)$ attains the value $\rho _S$. It is not required that $\rho _V$ vanishes on an interval: the efficiency of the vaccine translates into $\rho _V$ being *”very small”* for a *“very long”* time

Qualitatively, in the case of a vaccine consisting of a single dose, the function $\rho _V$ can be chosen, for instance, as depicted in Fig. 6. In the case of a vaccine consisting of 2 shots, a possible behavior of $\tau \mapsto \rho _V (\tau )$ is in Fig. 7.

Quantitatively, in both cases, the function $\rho _V$ depends on parameters specific to the vaccine under consideration.

In (2.4), a key role is played by the function $p = p (t, S, V, I, R)$. It describes the vaccination strategy, quantifying how many susceptible individuals are dosed at time *t*. Analytically, remark that the dependence of *p* on the variables *S*, *V*, *I*, *R* may well be of a *functional* nature, in the sense that *p* may depend, for instance, on time integrals of the functions *S*, *V*, *I*, *R*, see (2.12).

Several statistics on the solutions to (2.4) are of interest. First, the total number of casualties ${\mathcal {D}} (t_0,T)$ between time $t_0$ and time *T* (with $t_0 < T$) clearly equals the variation in the total number of individuals between times $t_0$ and *T*. It can be computed as

2.6 $${\mathcal{D}}(t_{0} ,T) = \left( {S(t_{0} ) + \int\limits_{0}^{{T_{V} }} V (t_{0} ,\tau ){\text{d}}\tau + I(t_{0} ) + \int\limits_{0}^{{T_{R} }} R (t_{0} ,\tau ){\text{d}}\tau } \right) - \left( {S(T) + \int\limits_{0}^{{T_{V} }} V (T,\tau ){\text{d}}\tau + I(T) + \int\limits_{0}^{{T_{R} }} R (T,\tau ){\text{d}}\tau } \right) = \int\limits_{{t_{0} }}^{T} \mu I(t){\text{d}}t.$$

An estimate of the cost of the vaccination campaign is given by the total number of vaccines dosed between time $t_0$ and time *T*, that is

2.7 $$\begin{aligned} {\mathcal {V}} (t_0,T) = \int _{t_0}^T p\left( t, S (t), V (t), I (t), R (t)\right) {\textrm{d}{t}} \,. \end{aligned}$$

A common index used to measure the virus propagation is the basic reproduction number (Murray 2002, Sect. 10.2), which is here computed as

2.8 $$\begin{aligned} {\mathcal {R}}_0 (t) = \dfrac{\rho _S \, S + \int _0^{T_V} \!\! \rho _V (\tau ) \, V (t,\tau ) {\textrm{d}{\tau }}+ \int _0^{T_R} \!\!\rho _R (\tau ) \, R (t,\tau ) {\textrm{d}{\tau }}}{\theta +\mu } \end{aligned}$$

since we have the equivalences

$$\begin{aligned} {\mathcal {R}}_0 (t)> 1 \iff \dot{I} (t) > 0 \quad \text{ and } \quad {\mathcal {R}}_0 (t)< 1 \iff \dot{I} (t) < 0 \,. \end{aligned}$$

Remark that the above expression of ${\mathcal {R}}_0 (t)$ does not require the knowledge of the number of infected *I*(*t*).

### Comparing Vaccination Strategies

Our aim in the integrations below is to stress qualitative features of the model (2.4). Quantitative data are presented to allow the reader to reproduce the results. Where helpful, we provide references coherent with the quantitative choices adopted, bearing in mind that several measurements are currently being improved and updated in the literature. Nevertheless, it may help the reader to consider time as measured in days, while *S*(*t*), *I*(*t*), $\int V (t,\tau ) {\textrm{d}{\tau }}$ and $\int R (t,\tau ) {\textrm{d}{\tau }}$ are percentages, since the total initial population is throughout fixed to 100.

The numerical algorithm adopted is described in Appendix B.

**The Reference Situation** We take as reference situation the spreading of virus with no vaccination, described by (2.4) with $p \equiv 0$ and with the following choices, which do not pretend to be quantitatively fully justified by the available data:

2.9 $$\begin{array}{*{20}c} {\rho _{S} = 1.0 \times 10^{{ - 3}} } & {\vartheta = 4.0 \times 10^{{ - 2}} } & {\mu = 2.0 \times 10^{{ - 3}} } \\ {\rho _{V}^{ - } = 1.0 \times 10^{{ - 5}} } & {\rho _{V} (\tau ) = \rho _{V}^{ - } + (\rho _{S} - \rho _{V}^{ - } )\;\Psi (\tau /T_{V} )} & {T_{V} = 180} \\ {\rho _{R}^{ - } = 2.0 \times 10^{{ - 5}} } & {\rho _{R} (\tau ) = \rho _{R}^{ - } + (\rho _{S} - \rho _{R}^{ - } )\;\Phi (\tau /T_{R} )} & {T_{R} = 180} \\ \end{array}$$

where $\Psi$ is as in (2.5) and $\Phi$ is as in (2.3). Since $\theta /\mu = 20$, see Russo et al. (2021), the above choice says that for an infected individual it is 20 times easier to recover than to die, corresponding to a mortality slightly lower than $5\%$. The different partial immunizations provided by the vaccine or by the recovery are described through the maps $\rho _V$ and $\rho _R$, displayed in Fig. 1, right. In the literature, available data keep being updated: with the present choice (2.9), the vaccine is more efficient than the recovering, both because it leaves a lower probability to get infected and because it is effective for a longer time.

The initial datum is

2.10 $$\begin{aligned}{} & {} S (0) = 95 \,,\quad V (0,\tau ) = 0 \text{ for } \tau \in [0, T_V] \,,\quad I (0) = 5 \,,\nonumber \\ {}{} & {} \quad R (0,\tau ) = 0 \text{ for } \tau \in [0, T_R] \end{aligned}$$

meaning that at time $t=0$, the susceptibles are $95\%$ of the total population, $5\%$ is infected, none is vaccinated and none is among those who recovered.

In this reference situation, the casualties after time 730 (i.e., 2 years) amount to $15.1\%$ of the initial population. The numerical integration shows the insurgence of *“epidemic waves”* (Lemon and Mahmoud 2005), see Fig. 1, left.

In the examples below, we always let the vaccination campaign begin after time $t=30$, to allow for the onset of the virus spreading. This is described through the term $\chi _{[30, +\infty [} (t)$ in the vaccination strategy, see for instance (2.11). Note also that the time $T_V$ in (2.9) adopted below allows for multiple, up to 4, vaccinations of each single individual. Therefore, the number of doses may well exceed the total initial population, set to 100.

**Leaving a Non Vaccinated Percentage** Practical considerations based on the different attitudes (Wang et al. 2020) towards vaccines may induce or oblige to avoid dosing a given portion of the population. Here, we describe this situation through the vaccination strategy

2.11 $$p(t,S,V,I,R) = \chi _{{[30, + \infty [}} (t)\;\chi _{{]S_{*} , + \infty [}} (S)\quad {\text{where}}\quad \begin{array}{*{20}c} {\chi _{{[30, + \infty [}} (t) = \left\{ {\begin{array}{*{20}c} 0 & t & < & {30} \\ 1 & t & \ge & {30} \\ \end{array} } \right.} \\ {\chi _{{]S_{*} , + \infty [}} (S) = \left\{ {\begin{array}{*{20}c} 0 & S & \le & {S_{*} } \\ 1 & S & > & {S_{*} } \\ \end{array} } \right.} \\ \end{array}$$

meaning that when susceptibles are below the threshold value $S_*$, the vaccination campaign stops. Note that, in the framework resulting from (2.4) to (2.11), we do not impose that the non vaccinated individuals are always the same.

As is to be expected, the higher the threshold $S_*$, the higher the resulting number of casualties. However, we remark that when the threshold percentage of non vaccinated gets near to $10\%$, the corresponding number of casualties sharply increases, see Fig. 4.

While it is somewhat arbitrary to choose a specific percentage where this sharp increase begins, this behavior partly justifies the term *“herd immunity”* (Randolph and Barreiro 2020), commonly used.

The actual computed values are in Table 1, where the case $S_* = 100$ corresponds to the reference situation above.

**Table 1:** Casualties and vaccinations in the solution to (2.4)–(2.9)–(2.10) corresponding to different values of $S_*$ in (2.11). See also Fig. 4

$S_*$	0	5	10	15	20	25	30	35	40	45	50	100
Deaths	3.59	3.99	4.60	5.71	8.34	12.5	13.8	14.3	14.5	14.6	14.8	15.1
Doses	326	298	267	227	154	61.3	30.5	17.7	13.8	11.3	8.78	0.00

**Automatic Feedback Based on** ${\mathcal {R}}_0 (t)$: Rather than a systematic full speed vaccination campaign, as considered in the preceding paragraph, one may consider a feedback strategy relying on the index ${\mathcal {R}}_0$ defined in (2.8). With the same notation as in (2.11), we set

2.12 $$\begin{aligned} p (t,S, V, I, R) = p_* \;\; \chi _{[30, +\infty [} (t) \;\; \chi _{]0,+\infty [} (S) \;\; \chi _{]r_*,+\infty [} ({\mathcal {R}}_0) \end{aligned}$$

meaning that at time *t*, with $t > 30$, the campaign proceeds dosing $p_*$ individuals per day, as soon as there are susceptibles (i.e., $S (t) > 0$) and ${\mathcal {R}}_0 (t)$ exceeds the threshold $r_*$.

This feedback strategy allows for a qualitative result, which is *independent* of the specific data and parameters chosen. Indeed, assume the strategy *p* in (2.12) is assigned so that ${\mathcal {R}}_0$ is stabilized to $r_*$ after time $t_*$, i.e., ${\mathcal {R}}_0 (t) = r_*$ for $t \in [t_*, T]$ for a large *T*. We can clearly assume that $t_* > T_V$ and $t_* > T_R$. Then, by (2.8), the solution to model (2.4) for $t \in [t_*, T]$ satisfies

2.13 $$\begin{aligned} \left\{ \begin{array}{rcl} \displaystyle I (t) &{} = &{} \displaystyle I (t_*) \; \exp \left( (r_*-1) (\theta +\mu ) (t-t_*)\right) \\ \displaystyle R (t,\tau ) &{} = &{} \displaystyle \theta \, I (t-\tau ) \; \exp \left( -\int _{t-\tau }^t \rho _R \, I (s) {\textrm{d}{s}}\right) \end{array} \right. \end{aligned}$$

see Lemma A.2. As expected, in the case $r_* = 1$, stabilizing ${\mathcal {R}}_0 (t)$ for $t \in [t_*,T]$, also *I* is stabilized at the value $I_* = I (t_*)$, and $R (t,\tau ) = \theta \, I_* \, e^{-\rho _R I_* \, \tau }$ is independent of *t*. Note that casualties, defined in (2.6), grow linearly with time, proving that *T* is necessarily bounded, its largest possible value corresponding to when all individuals die.

For arbitrary values of $r_*$, the former relation in (2.13) immediately gives for $r_* \ne 1$,

2.14 $$\begin{aligned} {\mathcal {D}} (t_*,T) = \dfrac{1}{1-r_*} \; \dfrac{\mu }{\theta +\mu } \left( 1 - e^{- (1-r_*) (\theta +\mu ) (T-t_*)} \right) I (t_*) \,. \end{aligned}$$

Thus, for the disease to disappear, it is necessary to stabilize ${\mathcal {R}}_0 (t)$ at a value $r_*$ strictly lower than 1. However, this condition is clearly not sufficient: one should also require that ${\mathcal {D}} (t_*,T)$ in (2.14) does not exceed the number of living individuals at time $t_*$.

**Fig. 8:**
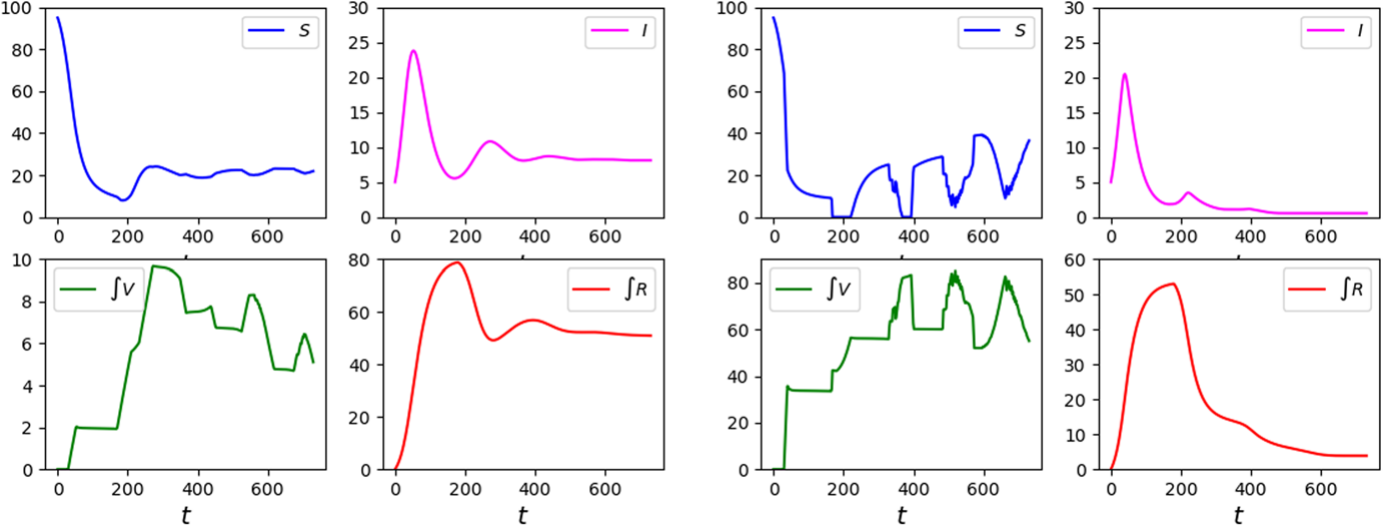
Solutions to (2.4)–(2.9)–(2.10) with strategy (2.12) with $r_* = 1.00$ and, left, $p_* = 0.1$ while, right, $p_* = 4.0$. Note that in both cases *I* is stabilized at a strictly positive value, left $I_* \approx 7$ and, right, $I_* \approx 1.5$, causing casualties to keep increasing, see Table 2. (The left and right scales in the lower diagrams of $\int V$ differ.)

**Fig. 9:**
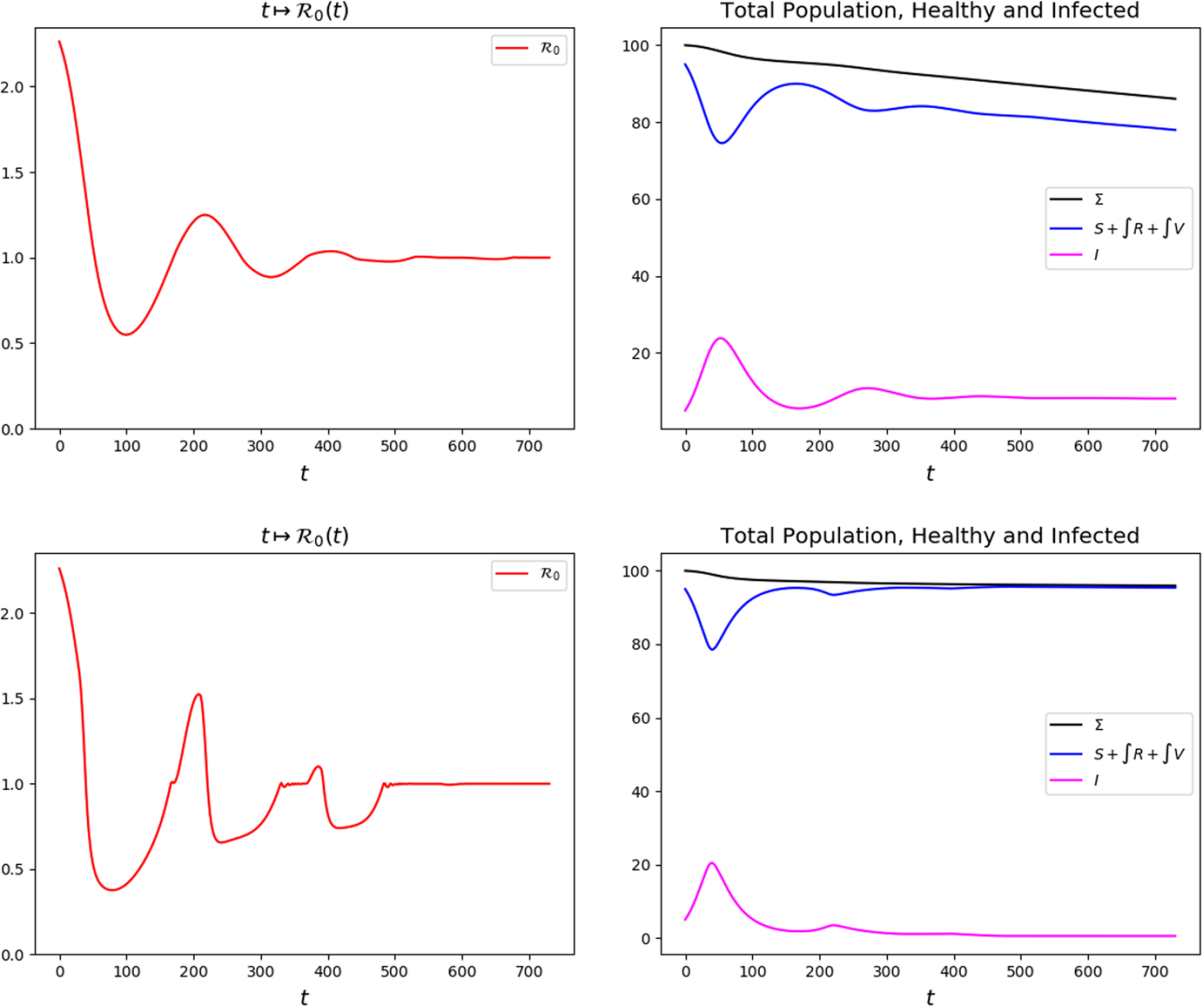
Solutions to (2.4)–(2.9)–(2.10) and the resulting values of ${\mathcal {R}}_0 (t)$ under strategy (2.12), $r_* =1.00$ with, above, $p_* = 0.1$ and, below, $p_* = 4.0$. The lower vaccination rate above causes a stabilization of *I* at a higher value and, hence, a higher mortality

The integrations in Fig. 8 confirm that stabilizing at ${\mathcal {R}}_0 (t) = 1$ does not stop the spreading of the disease, as also shown in Table 2. When ${\mathcal {R}}_0 (t) = 1$, a sort of *“dynamic equilibrium”* is onset, so that, for large *t*, the maps $t \rightarrow I (t)$ and $t \rightarrow \int R (t,\tau ) {\textrm{d}{\tau }}$ are approximately constant, while $t \rightarrow S (t)$ and $t \rightarrow \int V (t,\tau ){\textrm{d}{\tau }}$ have oscillations that approximately balance each other, so that their sum keep diminishing at a rate approximately $\mu \, I (t_*)$, see Fig. 9.

Somewhat surprisingly, two situations arise where a higher vaccination speed allows for a faster reduction of the infected and infectious population, so that – on the time interval considered – the resulting number of casualties is lower than that obtained after a higher but slower vaccination campaign, see the bold data in Table 2.

**Table 2:** Casualties (2.6), left, and total number of vaccinations (2.7), right, corresponding to the strategy (2.12) in (2.4)–(2.9)–(2.10). The bold data correspond to an unusual situation where less doses allow for less casualties, see also Fig. 10

Deaths		Threshold $r_*$			Doses		Threshold $r_*$		
${\mathcal {D}} (0, 730)$		0.25	0.50	1.00	${\mathcal {V}} (0,730)$		0.25	0.50	1,00
Speed $p_*$	0.10	12.1	12.1	13.9	Speed $p_*$	0.10	70.0	69.0	27.8
	0.50	**4.65**	4.89	10.2		0.50	**291**	287	109
	1.00	3.63	3.98	8.61		1.00	325	315	145
	1.50	3.18	3.53	7.40		1.50	336	328	173
	2.00	2.94	3.22	6.44		2.00	341	334	195
	2.50	2.79	2.99	5.66		2.50	345	338	214
	3.00	2.68	2.80	5.07		3.00	348	345	229
	3.50	2.60	2.64	**4.45**		3.50	350	349	**248**
	4.00	2.53	2.55	4.07		4.00	351	351	261

**Fig. 10:**
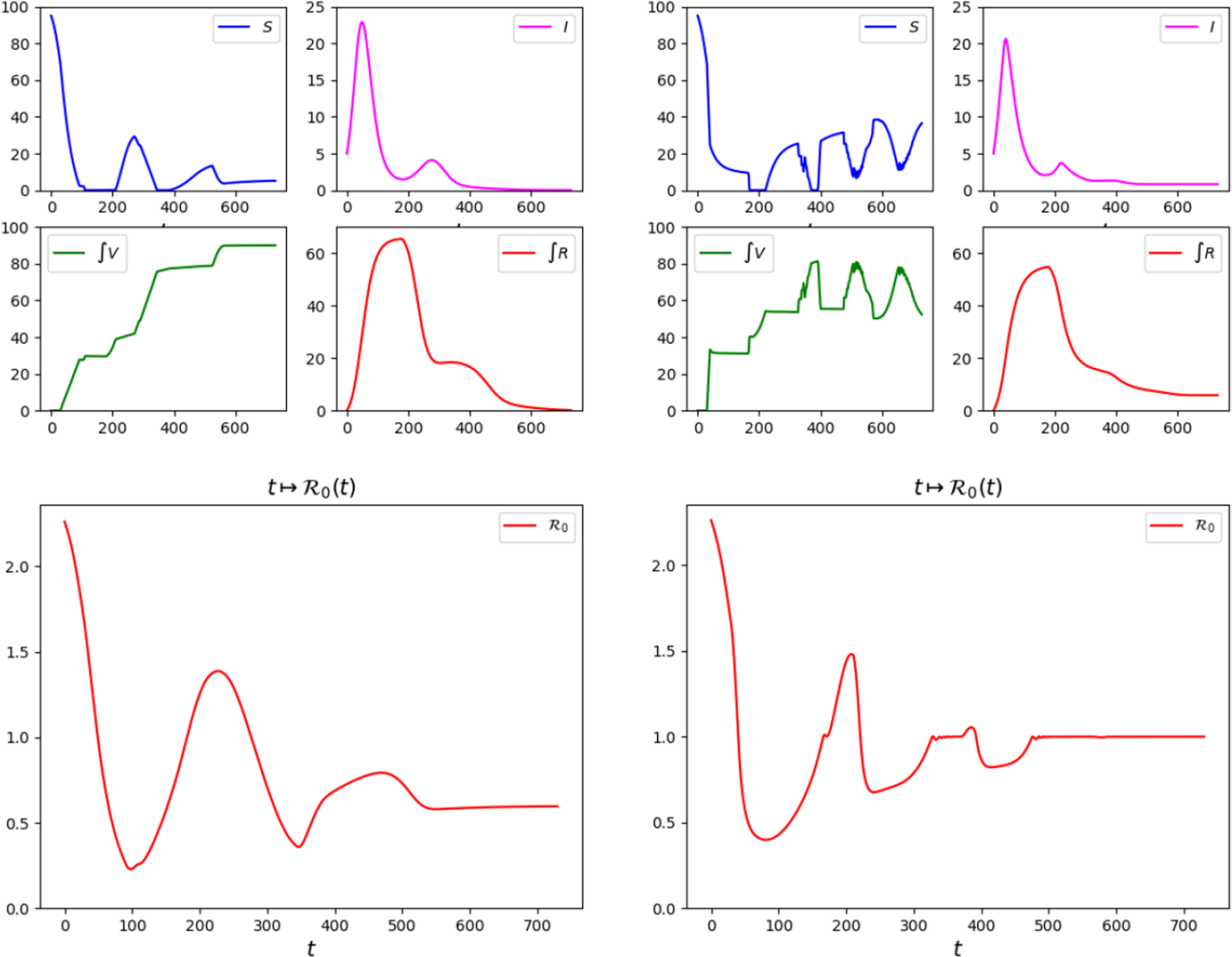
Solutions to (2.4)–(2.9)–(2.10) with strategy (2.12) and the resulting values of ${\mathcal {R}}_0 (t)$ corresponding to the bold data in Table 2: left, $p_* = 0.50$ and $r_* = 0.25$; right, $p_*=3.50$ and $r_* = 1.00$; above, the solutions and, below, the value of ${\mathcal {R}}_0 (t)$ along the solutions. The higher vaccination rate in the 2 diagrams on the right allows to get a lower number of casualties with a lower number of doses. In subsequent times, the lower value of ${\mathcal {R}}_0 (t)$ ensures that the choice on the left will be more effective in reducing the mortality

More precisely, a higher vaccination speed allows for a faster reduction of the *I* population and to quickly dose all the *S* individuals or lower ${\mathcal {R}}_0$ below the desired threshold, see Fig. 10.

On the other hand, the lower value of ${\mathcal {R}}_0 (t)$ obtained with the slower campaign ensures that in subsequent times this strategy results in being more effective in lowering casualties.

**Infinite Time Immunization** Model (2.4) can describe also the situation where the immunization provided by the vaccine and/or acquired after recovering lasts for ever. In the case $T_V \rightarrow +\infty$, system (2.4) becomes

2.15 $$\begin{aligned} \left\{ \begin{array}{l@{}} \displaystyle \dot{S} = - \rho _S \, I \, S + R (t, T_R) - p (t, S, V, I, R) \\ \displaystyle \partial _t V + \partial _\tau V = - \rho _V \, V \, I \quad\tau \in \mathopen [0, +\infty \mathclose [ \\ \displaystyle \dot{I} = \left( \rho _S \, S + \int _0^{+\infty } \!\! \rho _V (\tau ) \, V (t,\tau ) {\textrm{d}{\tau }}+ \int _0^{T_R} \!\!\rho _R (\tau ) \, R (t,\tau ) {\textrm{d}{\tau }}- (\theta + \mu ) \right) I \qquad \\ \displaystyle \partial _t R + \partial _\tau R = - \rho _R \, R \, I\quad \tau \in [0, T_R] \\ \displaystyle V (t, 0) = p(t,S, V, I, R) \\ \displaystyle R (t, 0) = \theta \, I \end{array} \right. \end{aligned}$$

where it is clear that individuals that entered the *V* population will remain therein. An entirely similar system can be used to describe the case $T_R \rightarrow +\infty$.

**Fig. 11:**
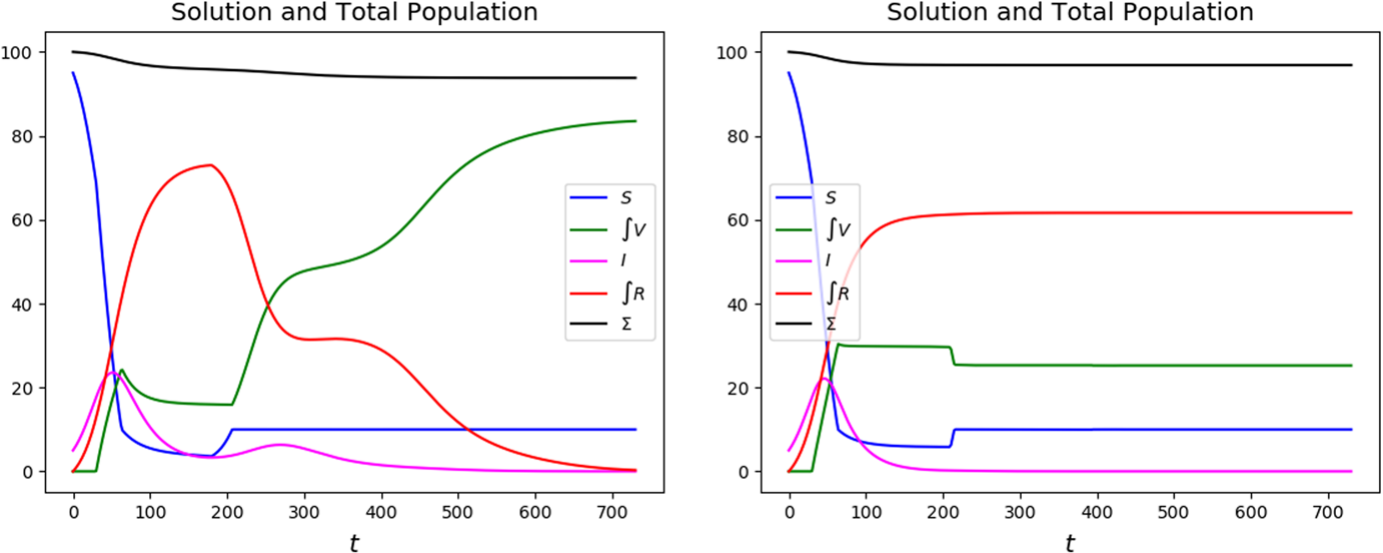
In these integration we used parameters (2.9), the data (2.10) and the strategy (2.11) with $S_*=10$. Left, the case (2.15) where $T_V \rightarrow +\infty$ with $T_R = 180$. Right, the case $T_R\rightarrow +\infty$ with $T_V = 180$. Note that epidemic waves essentially disappeared, quite quickly in the case $T_R \rightarrow +\infty$ on the right

In the integrations below, we keep using the choice (2.9), the data (2.10) and the strategy (2.11) with $S_*=10$. The resulting integrations, displayed in Fig. 11, show an evident stabilization effect induced by the infinite duration of the immunization.

**Fig. 12:**
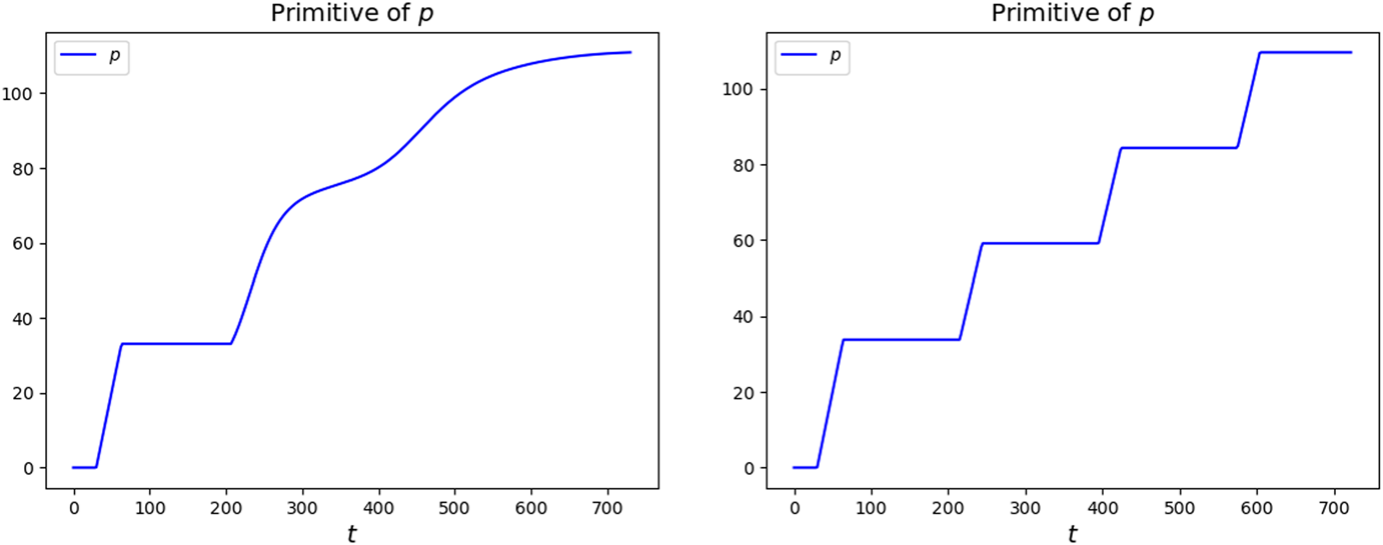
Distribution function $t \mapsto \int _0^t p (\tau ) {\textrm{d}{\tau }}$ of the vaccination strategies $p = p (t)$ in the integrations corresponding to parameters (2.9), data (2.10) and strategy (2.11) with $S_*=10$. Left, the case (2.15) where $T_V \rightarrow +\infty$ with $T_R = 180$. Right, the case $T_R\rightarrow +\infty$ with $T_V = 180$. Note that, on the right, the vaccination campaign is activated as soon as *S* individuals are available, i.e., when vaccinated get back to be susceptible

Indeed, epidemic waves are rather quickly smeared out, in particular in the case $T_R \rightarrow +\infty$. As soon as individuals enter the *R* population, they will (almost) never leave it, while all susceptible individuals are vaccinated as soon as the effect of the previous vaccination disappears, see Fig. 12 on the right.

## Concurrent Vaccines

We now consider the case of a vaccination campaign based on the concurrent use of two different vaccines, so that vaccinated individuals enter the population $V_1$ or $V_2$ depending on whether they were dosed with vaccine 1 or with vaccine 2. Thus, $V_1 (t,\tau )$, respectively $V_2 (t,\tau )$, measures the amount of individuals at time *t* that were dosed at time $t-\tau$ with vaccine 1, respectively with vaccine 2. We also need to introduce the controls specifying the speed at which the 2 vaccines are dosed. The equation for the *S* population then reads:

3.1 $$\begin{aligned} \begin{array}{rcl} \dot{S} &{} = &{} -\rho _S \, I \, S + V_1 (t,T_1) + V_2 (t,T_2) + R (t, T_R) \\ &{} &{} - p_1 (t, S, V_1, V_2, I, R) - p_2 (t, S, V_1, V_2, I, R) \end{array} \end{aligned}$$

where we used obvious modification of the notation in (2.4). The above equation also prescribes that at time $T_1$, individuals in the $V_1$ population get back to be susceptible, and similarly for $T_2$.

Extending (2.4), for the $V_1$, $V_2$ and *R* populations we obtain

3.2 $$\begin{aligned} \left\{ \begin{array}{@{}ll@{}} \displaystyle \partial _t V_1 + \partial _\tau V_1 = -\rho _1 (\tau ) \, V_1 (t,\tau ) \, I &{} \tau \in [0, T_1] \\ \displaystyle \partial _t V_2 + \partial _\tau V_2 = -\rho _2 (\tau ) \, V_2 (t,\tau ) \, I &{} \tau \in [0, T_2] \\ \displaystyle \partial _tR + \partial _\tau R = -\rho _R (\tau ) \, R (t,\tau ) \, I &{} \tau \in [0, T_R] \\ \displaystyle V_1 (t, 0) = p_1 (t, S, V_1, V_2, I, R) \\ \displaystyle V_2 (t, 0) = p_2 (t, S, V_1, V_2, I, R) \\ \displaystyle R (t, 0) = \theta \, I \end{array} \right. \end{aligned}$$

where, similarly to the previous section, the three time scales $[0,T_1]$, $[0,T_2]$ and $[0, T_R]$ are entirely independent.

Finally, the *I* population varies partly due to the propagation of the infection and partly due to infected individuals recovering or dying:

3.3 $$\begin{aligned} \begin{array}{rcl} \dot{I} &{} = &{} \displaystyle \rho _S \, S \, I + \int _0^{T_1} \rho _1 (\tau ) \, V_1 (t,\tau ) {\textrm{d}{\tau }}\, I + \int _0^{T_2} \rho _2 (\tau ) \, V_2 (t,\tau ) {\textrm{d}{\tau }}\, I \\ &{} &{} \displaystyle + \int _0^{T_R} \rho _R (\tau ) \, R (t,\tau ) {\textrm{d}{\tau }}\, I - \theta \, I - \mu \, I \,. \end{array} \end{aligned}$$

The natural extension of (3.1)–(3.2)–(3.3) when *k* different vaccines are available reads

3.4 $$\begin{aligned} \left\{ \begin{array}{l} \displaystyle \dot{S} = -\rho _S \, I \, S + \sum _{i=1}^k V_i (t,T_i) + R (t, T_R) - \sum _{i=1}^kp_i (t, S, V, I, R) \\ \displaystyle \partial _tV_i+ \partial _\tau V_i = -\rho _i (\tau ) \, V_i (t,\tau ) \, I\quad \tau \in [0, T_i] \qquad i=1 ,\ldots , k \\ \displaystyle \dot{I} = \rho _S \, I \, S + \left( \sum _{i=1}^k \int _0^{T_i} \rho _i (\tau ) \, V_i (t,\tau ) {\textrm{d}{\tau }}+ \int _0^{T_R} \rho _R (\tau ) \, R (t,\tau ) {\textrm{d}{\tau }}\right) I - \theta \, I - \mu \, I \\ \displaystyle \partial _tR + \partial _\tau R = -\rho _R (\tau ) \, R (t,\tau ) \, I\quad \tau \in [0, T_R] \\ \displaystyle V_i (t, 0) = p_i (t, S, V, I, R) \\ \displaystyle R (t, 0) = \theta \, I \,. \end{array} \right. \end{aligned}$$

Also in this general case, the index ${\mathcal {R}}_0 (t)$ can be defined as

3.5 $$\begin{aligned} {\mathcal {R}}_0 (t) = \dfrac{\rho _S \, S + \sum _{i=1}^k \int _0^{T_i} \!\! \rho _i (\tau ) \, V_i (t,\tau ) {\textrm{d}{\tau }}+ \int _0^{T_R} \!\!\rho _R (\tau ) \, R (t,\tau ) {\textrm{d}{\tau }}}{\theta +\mu } \end{aligned}$$

and identifies the times where $\dot{I}$ is positive or negative, without explicitly requiring knowledge of *I*(*t*).

### Comparing the Effects of Different Vaccines

**Fig. 13:**
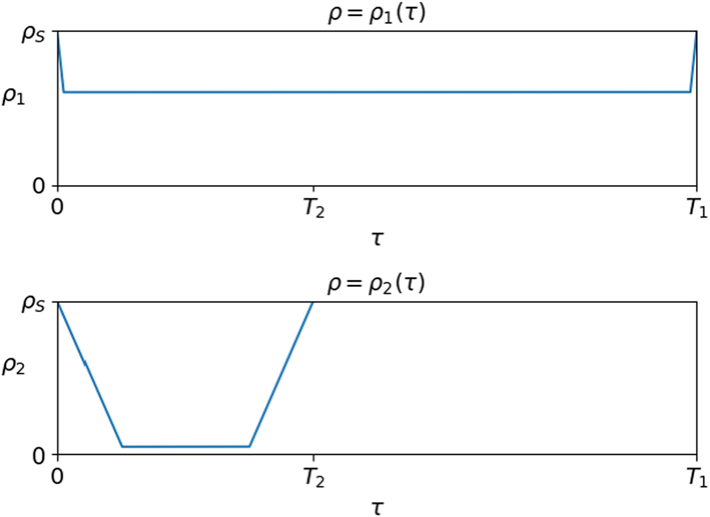
Characterizations of the vaccines used in the integrations of (3.4) with $k=2$ and the choices (2.3)–(2.4)–(2.9)–(2.10). Vaccine 1 provides a weak protection almost immediately, while vaccine 2 is more protective but after a while and for a shorter time

In the integrations of this paragraph we keep using the choices (2.4)–(2.9)–(2.10), so that the reference situation with no vaccination campaign is the one discussed in Sect. 2.1 and illustrated in Fig. 1. We introduce 2 vaccines, say 1 and 2, characterized by the diagrams in Fig. 13, see also (3.6).

3.6 $$\begin{gathered} \begin{array}{*{20}c} {\rho _{1} (\tau ) = 6.0 \times 10^{{ - 6}} \;{\text{for}}\;\tau \in [3,297]} \\ {T_{1} = 300} \\ \end{array} \hfill \\ \begin{array}{*{20}c} {\rho _{2} (\tau ) = 5.0 \times 10^{{ - 7}} \;{\text{for}}\;\tau \in [30,90]} \\ {T_{2} = 120} \\ \end{array} \hfill \\ \end{gathered}$$

We consider the strategies

(11) Vaccine 1 is used throughout, from time $t=30$ on, while Vaccine 2 is not used.
(12) Vaccine 1 is used for $t \in [30, 380]$, while Vaccine 2 is used for $t \in [380, 730]$.
(21) Vaccine 2 is used for $t \in [30, 380]$, while Vaccine 1 is used for $t \in [380, 730]$.
(22) Vaccine 2 is used throughout, from time $t=30$ on, while Vaccine 1 is not used.
(1/2) Both vaccines are used throughout, with the same number of doses.

In the present setting we are assuming that vaccinated individuals that get back to be susceptibles are vaccinated as soon as possible. Therefore, it is intuitive that Vaccine 2 results in being the best choice, as shown in Table 3.

**Table 3:** Statistics on the solutions to (3.4) with $k=2$ and the choices (2.9)–(2.10)–(3.6) corresponding to the strategies outlined in Sect. 3.1. The leftmost column refers to the reference solution where no vaccination takes place, see Sect. 2.1

Strategy	ref.	(11)	(12)	(21)	(22)	(1/2)
Deaths	15.1	11.4	7.20	4.26	3.92	5.78
1 Doses	0.00	187	125	131	0.00	159
2 Doses	0.00	0.00	151	215	480	159
Doses Tot.	0.00	187	275	346	480	318

Indeed, in the present framework, once an individual is vaccinated with a Vaccine 1, he/she can not be vaccinated using the more efficient Vaccine 2 as long as the first immunization is, though only poorly, effective. This also explains the different outcomes of the strategies (12) and (21). Note also that strategy (22) allows to dose $80\%$ of the initial population 5 times, see Table 3.

## Continuous and Discrete Age Structures

Age differences can play a significant role in the reaction of individuals to the infection. We thus extend our framework to account also for age differences. First, we insert a continuous age structure, later in Sect. 4.1 we consider discrete age classes. In the first case $a \in {\mathbb {R}}_+$ is a continuous variable and the convective terms $\partial _a S, \partial _a V, \partial _a I, \partial _a R$ in (4.1) describe the aging of the individuals in the population *S*, *V*, *I*, *R*. In the latter case, *a* is a discrete variable ranging in the finite set of the age classes considered and no aging term is present, see (4.3). The former approach seems more accurate, but on short time intervals the second is a usual and acceptable simplification.

For simplicity, we detail the age structured version of (2.4) corresponding to only one vaccine. The extension of the *k* vaccines case (3.4) being only technically more intricate. We thus obtain:

4.1 $$\begin{aligned} \left\{ \begin{array}{l@{}} \displaystyle \partial _t S + \partial _a S = - \rho _S \, I \, S + V \left( t, a, T_V (a)\right) + R \left( t, a, T_R (a)\right) - p (t, a, S, V, I, R) \\ \displaystyle \partial _t V + \partial _a V + \partial _\tau V = - \rho _V \, V \, I \quad\tau \in [0, T_V (a)] \\ \displaystyle \partial _t I + \partial _a I = \rho _S \, S \, I + \int _0^{T_V} \!\! \rho _V (a, \tau ) \, V (t, a, \tau ) {\textrm{d}{\tau }}\, I + \int _0^{T_R} \!\!\rho _R (a, \tau ) \, R (t, a, \tau ) {\textrm{d}{\tau }}\, I \\ \qquad \qquad \qquad - \theta \, I - \mu \, I \\ \displaystyle \partial _t R + \partial _a R + \partial _\tau R = - \rho _R \, R \, I \quad \tau \in [0, T_R (a)] \\ \displaystyle V (t, a, 0) = p(t,a, S, V, I, R) \\ \displaystyle R (t, a, 0) = \theta \, I \,. \end{array} \right. \end{aligned}$$

Note that here all effects of vaccines are age dependent. The immunization time provided by the vaccine is $T_V = T_V (a)$ and, similarly, also the immunization ensured by recovering from the disease is age dependent: $T_R = T_R (a)$. Remark that (4.1) is able to take into consideration the different effectiveness of the vaccine at different ages, thanks to the dependence of $\rho _V$ also on *a*: $\rho _V = \rho _V (a, \tau )$. Similarly, in (4.1) also the recovery rate $\theta$ depends on the age, i.e., $\theta = \theta (a)$, as well as the mortality rate, $\mu = \mu (a)$.

As usual in age structured models, further boundary conditions need to be supplemented, taking care of the newborns, such as

4.2 $$\begin{aligned} S (t, 0) = b (t) \,,\qquad V (t,0,\tau ) = 0 \,,\qquad I (t,0) = 0 \,,\qquad R (t,0,\tau ) = 0 \,, \end{aligned}$$

where *b*(*t*) is the time dependent natality. Other natality terms can be considered, depending, for instance, on the total amount of susceptibles.

However, typically, the use of a pandemic model may be of interest on time intervals far smaller than the average life span of individuals. Therefore, it is convenient to consider a fixed number, say *m*, of age classes. As a consequence, we have *m* different populations of susceptibles, of vaccinated, infected and recovered, obtaining the mixed multiscale system

4.3 $$\begin{aligned} \left\{ \begin{array}{l} \displaystyle \dot{S}_a = - \left( \sum _{\alpha =1}^m \rho _S^{a,\alpha } \, I_\alpha \right) S_a + V_a (t,T_V^a) + R_a (t, T_R^a) - p_a (t, S, V, I, R) \\ \displaystyle \partial _t V_a + \partial _\tau V_a = - \left( \sum _{\alpha =1}^m \rho _V^{a,\alpha } \, I_\alpha \right) V_a\quad \tau \in [0, T_V^a] \\ \displaystyle \dot{I}_a = \left( \sum _{\alpha =1}^m \rho _S^{a,\alpha } \, I_\alpha \right) S_a + \left( \sum _{\alpha =1}^m I_\alpha \int _0^{T_V^a} \rho _V^{a,\alpha } (\tau ) \, V_a (t,\tau ) {\textrm{d}{\tau }}\right) \\ \qquad \qquad \displaystyle + \left( \sum _{\alpha =1}^m I_\alpha \int _0^{T_R^a} \rho _R^{a,\alpha } (\tau ) \, R_a (t,\tau ) {\textrm{d}{\tau }}\right) - \theta _a \, I_a - \mu _a \, I_a \\ \displaystyle \partial _t R_a + \partial _\tau R_a = - \left( \sum _{\alpha =1}^m \rho _R^{a,\alpha } \, I_\alpha \right) R_a \quad\tau \in [0, T_R^a] \\ \displaystyle V_a (t, 0) = p_a(t,S, V, I, R) \\ \displaystyle R_a (t, 0) = \theta _a \, I_a \end{array} \right. a=1, \ldots , m\,. \end{aligned}$$

Above, the terms $\rho _S^{a,\alpha }$, $\rho _V^{a,\alpha }$ and $\rho _R^{a,\alpha }$ quantify the spreading of the virus between the age class *a* and the age class $\alpha$ in the populations *S*, *V* and *R*.

In (4.3), differently from what happens in (4.1), the total number of individuals in the age class *a* may vary only due to the mortality in that class, i.e.,

4.4 $$\begin{aligned} \dfrac{{\textrm{d}{~}}}{{\textrm{d}{t}}} \left( S_a (t) + \int _0^{T_V^a} V_a (t,\tau ) {\textrm{d}{\tau }}{} + I_a (t) + \int _0^{T_R^a} R_a (t,\tau ) {\textrm{d}{\tau }}{} \right) = -\mu _a \, I_a (t) \,, \end{aligned}$$

so that infection propagates among individuals of different classes, but no individual changes its age class.

The introduction of an index similar to ${\mathcal {R}}_0 (t)$ is formally possible, but the resulting expression necessarily explicitly depends on $I_a (t)$.

### Comparing Age Dependent Vaccination Strategies

We limit the numerical integrations of (4.3) to the case of only 2 classes, say the *young* one (indexed with 1) and the *old* one (2). For a different approach to the modeling of 2 age classes, refer for instance to Verrelli and Della Rossa (2021).

**The Reference Situation** Consider first the case where no vaccination campaign takes place. On the basis of a qualitative approach as in Sect. 2.1, we choose the following set of parameters:

4.5 $$\begin{array}{*{20}c} {\rho _{S}^{{11}} = 3.0 \times 10^{{ - 3}} } & {\rho _{S}^{{12}} = 1.0 \times 10^{{ - 3}} } & {\rho _{S}^{{21}} = 2.0 \times 10^{{ - 3}} } & {\rho _{S}^{{22}} = 1.0 \times 10^{{ - 3}} } \\ {\theta ^{1} = 6.0 \times 10^{{ - 2}} } & {\theta ^{2} = 4.0 \times 10^{{ - 2}} } & {\mu ^{1} = 5.0 \times 10^{{ - 4}} } & {\mu ^{2} = 2.0 \times 10^{{ - 3}} }\\ {T_{R}^{1} = 180} & {T_{R}^{2} = 140} & {\rho _{R}^{ - } = 2.0 \times 10^{{ - 5}} } & \\ \end{array}$$

and we keep referring to the choices of $\Phi$ and $\Psi$ in (2.3) and (2.5), so that for $a,\alpha = 1,2$

4.6 $$\begin{aligned} \rho _V^{a,\alpha } (\tau ) = \rho _V^- + (\rho _S^{a,\alpha }-\rho _V^-) \; \Psi (\tau / T_V^{a,\alpha }) \quad \text{ and } \quad \rho _R^{a,\alpha } (\tau ) = \rho _R^- + (\rho _S^{a,\alpha }-\rho _R^-) \; \Phi (\tau / T_R^{a,\alpha }) \,. \end{aligned}$$

The above choices reflect the fact that class 2 individuals suffer a higher mortality ($\mu _2 = 4\, \mu _1$) and have a slower recovery ($\theta ^2= 0.67\, \theta ^1$). The two age classes differ also in the time scales, the younger ones having longer periods of (partial) immunization both after recovery and after vaccination. On the other hand, among class 1 individuals the virus spreads faster ($\rho ^{11}_S / \rho ^{22}_S = 3$).

Throughout, we carry the integrations up to a final time 730 (roughly corresponding to 2 years) and with the initial datum (for $a=1,2$)

4.7 $$\begin{aligned} S_1 (0) = 42\,,\quad S_2 (0) = 53\,,\quad V_a (0,\tau ) = 0 \,,\quad I_1 (0) = 1\,,\quad I_2 (0) = 4\,,\quad R_a (0,\tau ) = 0 \,. \end{aligned}$$

**Fig. 14:**
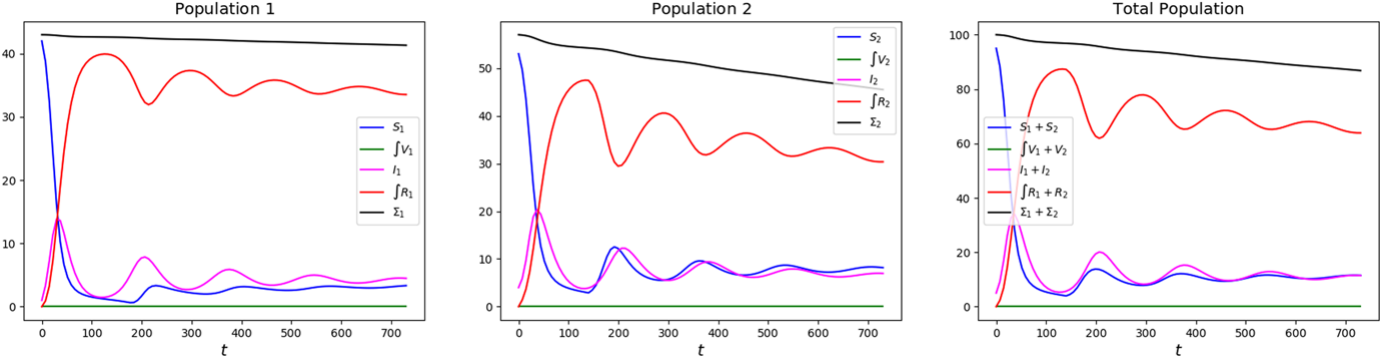
Solution to (4.3) with parameters (4.5)–(4.6) and initial datum (4.7) in the case no vaccination is dosed. Left, population 1; middle, population 2 and, right, their sum. In all diagrams, $\Sigma$ stands for the sum of all individuals of the considered age class(es)

The resulting evolution is displayed in Fig. 14.

In this reference situation, the casualties after time 730 (i.e., 2 years) amount to 1.67 in class 1, 11.5 in class 2, totaling to 13.2 (the total initial population being 100). Note the formation of *“epidemic waves”* (Lemon and Mahmoud 2005), Fig. 14.

In this framework, a variety of age dependent vaccination strategies can be adopted. Concerning the vaccine, following (2.9), we keep the following choices fixed:

4.8 $$\begin{aligned} \rho _V^{a,\alpha } (\tau ) = \rho _V^- + (\rho _S^{a,\alpha }-\rho _V^-) \; \Psi (\tau /T_V^a) \text{ for } a,\alpha =1,2 \qquad \text{ and } \quad \begin{array}{r@{\;}c@{\;}l} T_V^1 &{} = &{} 200 \\ T_V^2 &{} = &{} 160 \\ \rho _V^- &{} = &{} 1.0 \times 10^{-5} \end{array} \end{aligned}$$

where $\Psi$ is as in (2.5). Throughout, we let the vaccination campaign begin after time $t=30$. We consider below 4 instances:

Feedback: $p_a (t)$ is proportional to the number of infected individuals in class *a*, i.e., $p_a (t) = I_a (t) / \left( I_1 (t) + I_2 (t)\right)$ as long as $S_a (t) > 0$, for $a=1,2$.
Half–Half: $p_a (t) = 0.5$ as long as there are susceptibles in class *a*, i.e., $S_a (t)>0$, for $a=1,2$.
Class 2 First: for $t \in [30, 380]$, $p_1 (t) = 0$ and $p_2 (t) = 1$ as long as $S_2 (t)>0$; for $t \in [380, 730]$, $p_1 (t) = 1$ and $p_2 (t) = 0$ as long as $S_1 (t)>0$.
Class 1 First: for $t \in [30, 380]$, $p_1 (t) = 1$ and $p_2 (t) = 0$ as long as $S_1 (t)>0$; for $t \in [380, 730]$, $p_1 (t) = 0$ and $p_2 (t) = 1$ as long as $S_2 (t)>0$.

In all strategies, the total number of vaccines dosed per day is at most $1\%$ of the total initial population, as soon as the number of susceptibles is sufficiently high. In these examples, we also let vaccinated be dosed again as soon as they get back to be susceptible. The present framework clearly allows also to leave an amount of non vaccinated individuals, as in Sect. 2.1.

It is evident that Class 1 First is likely to be the least effective strategy, as it actually results from Table 4. Less intuitive is the fact that Class 2 First is only slightly better, in particular comparing the total number of casualties.

**Table 4:** Statistics on the solutions to (4.3) with parameters (4.5)–(4.6), initial datum (4.7) and with the vaccination strategies detailed in Sect. 4.1

Strategy	Reference	Feedback	Half–Half	Class 2 First	Class 1 First
1 deaths	1.67	0.620	0.630	1.24	1.35
2 deaths	11.5	3.52	3.66	7.47	8.51
Deaths tot.	13.2	4.14	4.29	8.71	9.86
1 doses	0.00	106	106	41.8	28.5
2 doses	0.00	206	203	87.0	84.4
Doses tot.	0.00	313	309	129	113

Surprisingly, Class 2 First results in a number of casualties in class 1 even lower than that resulting from strategy Class 1 First.

Moreover, in both Class 1 First and Class 2 First strategies, the rise of rather persisting epidemic waves is evident. In particular, in the latter case the final increase in the number of infected of both classes induces to expect a worsening of the situation in the long run.

Among the strategies considered, the one resulting most effective in containing casualties is the Feedback one. However, it is not easy to anticipate that, mainly due to the particular initial data chosen, it is only slightly better than the Half–Half one. Indeed, a feedback strategy is generally prone to provide better results than an open loop one.

It stems out of these examples that, in order to reduce the number of casualties, it is of key importance to bound the number of susceptibles, as a comparison between the graphs in Figs. 2 and 3 shows.

It is also worth noting that a *“weak”* vaccination campaign can lead to somewhat persistent epidemic waves. Indeed, compare the qualitative behavior of the maps $t \mapsto I_a (t)$ in the reference case (Fig. 14), in the successful cases Feedback or Half–Half to those corresponding to the strategies Class 1 First or Class 2 First (Fig. 2). The epidemic waves in the latter case appear to be quite persistent, while they fade out sooner in the former 2 cases. Indeed, the lack of any vaccination results in a high mortality that hinders the repeated formation of waves.

On the opposite, an efficient vaccination campaign quickly flattens the $S_a$ curve near to 0. A weak vaccination campaign still reduces the number of casualties but may be not sufficiently strong to eradicate the disease, which thus keeps propagating in waves.

## Parameters’ Choices in the Case of Covid–19

Here we present and justify possible *a priori* choices for the parameters and functions entering our framework on the basis of available measurements. A different approach might consist in fitting, *a posteriori*, the solutions to the above models to the measured evolution.

As is well known, the actual values of the parameters or functions $\rho _S, \rho _V, \rho _R, \theta , \mu$ appearing in the various models depend on possible normalizations of the total number of individuals. In the previous sections, for instance, we set the total initial population to 100. The time scales, defined for instance by $T_V$ and $T_R$ in the case of (2.4), can be directly deduced from the literature.

For simplicity, we refer to (2.4), the further parameters entering the other models can be evaluated similarly.

**Parameter** $\rho _S$: In any attempt to obtain real values from a predictive model, this parameter has to be taken as time dependent. Indeed, not only it heavily depends on the introduction of lockdown restrictions or on the season, the mere social awareness of the disease may significantly affect its value. As an example, we refer to (Law et al. 2020, Table 3), where time dependent values for $\rho _S$ (there denoted $z\, \beta _t$) are deduced from real data.

**Parameter** $T_V$ **and Function** $\rho _V = \rho _V (\tau )$: Here, by vaccination we intend the full treatment consisting of 2 injections, dosed sufficiently near so that there is no loss in the protection they provide. Therefore, we rely on a function $\rho _V$ of the type in Fig. 6, with $T_V$ being the duration of the immunization provided by the 2 doses. Due to the relatively short history Covid–19 vaccinations, this datum can only be inferred, see for instance (Baden et al. 2021; Polack et al. 2020). On the basis of Zenilman et al. (2021) and Whitley et al. (2021), it seems safe to assume $T_V = 180 \text{ days }$, i.e., 6 months, for the BNT162B2 mRNA vaccine as well as for the mRNA-1273 vaccine. A choice of $\rho _V^- = 0.05 \, \rho _S$ is realistic in the case of the BNT162b2 mRNA vaccine, see (Polack et al. 2020), while $\rho _V^- = 0.06 \, \rho _S$ seems justified by Baden et al. (2021) in the case of the mRNA-1273 vaccine.

**Parameter** $T_R$ **and Function** $\rho _R = \rho _R (\tau )$: The current literature provides different results about the duration of the immunization enjoyed by those who recovered from Covid–19. For instance, in Ripperger et al. (2020), the authors suggest for $T_R$ a value between 150 and 210 days. Assuming for the function $\rho _R = \rho _R (\tau )$ a shape like that in (2.3), we are left to estimate $\rho _R^-$, which measures the best level of protection provided by the recovery. In the data collected in Shrestha et al. (2021), none of the 1359 non vaccinated that recovered got infected. In Lumley et al. (2021), out of 11364 individuals that recovered, only 2 resulted infected according to a PCR test. Clearly, it is natural to expect that both $T_R$ and $\rho _R^-$ are significantly age dependent.

**Parameters** $\theta$ **and** $\mu$: Both these parameters should better be considered time dependent whenever simulations are meant to provide results on a scale of several months. Indeed, care protocols have been continuously updated since Covid–19 outbreak and new drugs have been introduced. As a reference, we recall that in it is suggested in Russo et al. (2021) that $\theta /\mu \approx 18.2$, obtained from statistics in the Milan (Italy) area between February 19th, 2020 and January 21st, 2021.

**Function** $p = p (t)$: This function quantifies how many vaccination are dosed per unit time (e.g. day). Clearly, it is time dependent and its value has been chosen according to different policies in different nations. Often, health care workers were given the highest priority with old or fragile individuals coming next. As reference values, we record that in Italy on August 4th 2021, 171565 individuals (i.e., about $0.29\%$ of the Italian population) received their first dose, while they were 5544 ($0.0093\%$) on November 1st, 2021, data taken from COVID-19 (2021).

## Conclusion

This paper introduces a framework for the multiscale modeling of a vaccination campaign in presence of a pandemic. Different concurrent vaccines can be considered, each of them is characterized by its own efficiency and provides an immunization whose level depends on the time since dosing. Different age classes can be considered to account for the dependence of mortality, infectivity, vaccine efficiency... on age. Within this framework, different vaccination strategies can be simulated and compared, in terms of casualties, number of infected individuals or number of vaccines dosed, for instance.

To our knowledge, a general theorem ensuring the well posedness of these models is still unavailable. We expect that this result is at reach along the lines in Colombo and Garavello (2021) and Colombo et al. (2022a). Also the search for an *“optimal”* vaccination strategy is still an open problem, however recent results are pointing in this direction; see for instance to Di Giamberardino et al. (2021), Keimer and Pflug (2020) and McQuade et al. (2021).

Aiming at quantitatively reliable forecasts by means of the present framework requires accurate knowledge of various data. In particular, the efficiency of vaccines, here quantified through the function $\rho _V = \rho _V (\tau )$ (or $\rho _V = \rho _V (\tau ,a)$), appears as quite difficult. In this connection, it looks promising to deal with the uncertainties intrinsic to these functions through the recent techniques in Albi et al. (2021a, b, c).

The present framework is quite flexible and several extensions are easily at reach. For instance, letting $\rho _S$ and/or $\rho _R$ depend explicitly also on time *t* may account for the insurgence of new virus mutations or strict lockdown policies. Spatial movements can be incorporated using exactly the same techniques as in (Colombo et al. 2022b, Sect. 6) or (Colombo et al. 2020, Formula (1). Gender differences only amount to introduce further distinctions among the unknown variables.

## Data Availability

Not applicable.

## Appendix A: Proof of (2.13) and (2.14)

### Lemma A.1

The solution to $$\left\{ {\begin{array}{*{20}c} {\partial _{t} u + \partial _{\tau } u = u\phi (t)} \\ {u(t,0) = \alpha \phi (t)} \\ \end{array} } \right.$$ is $u (t,\tau ) = \alpha \; \phi (t-\tau ) \, e^{\int _{t-\tau }^t \phi (s){\textrm{d}{s}}}$.

The proof is a straightforward computation, hence it is omitted.

### Lemma A.2

Assume that, for a suitable *p*, problem (2.4) admits a classical solution on ${\mathbb {R}}_+$ with ${\mathcal {R}}_0 (t)=r_*$ for all $t \in [t_*, +\infty [$, for a suitable $t_* > 0$ and with ${\mathcal {R}}_0$ as defined in (2.8). Then, (2.13) and (2.14) hold.

### Proof

The above assumptions ensure that *S*, *V*, *I*, *R* also solve for $t \ge t_*$ the mixed ODE–PDE problem

6.1 $$\begin{aligned} \left\{ \begin{array}{ll@{}} \displaystyle \dot{S} = - \rho _S \, I \, S + V (t,T_V) + R (t, T_R) - p (t, S, V, I, R) \\ \displaystyle \partial _t V + \partial _\tau V = - \rho _V \, V \, I &{} \tau \in [0, T_V] \\ \displaystyle \dot{I} = (r_*-1) \, (\theta +\mu ) \,I \\ \displaystyle \partial _t R + \partial _\tau R = - \rho _R \, R \, I &{} \tau \in [0, T_R] \\ \displaystyle V (t, 0) = p(t,S, V, I, R) \\ \displaystyle R (t, 0) = \theta \, I \end{array} \right. \end{aligned}$$

We then obtain a closed equation for *I*, namely $\dot{I} = (r_*-1) \, (\theta +\mu ) \,I$, whose solution is the first line in (2.13). Then, the initial–boundary value problem

$$\begin{aligned} \left\{ \begin{array}{l} \displaystyle \partial _t R + \partial _\tau R = - \rho _R \, R \, I \\ \displaystyle R (t, 0) = \theta \, I \end{array} \right. \end{aligned}$$

fits into Lemma A.1 with $u (t,\tau ) = R (t,\tau )$, $\phi (t) = -\rho _R \, I (t)$, $\alpha = -\theta /\rho _R$, proving the second line in (2.13).

Verifying (2.14) is now straightforward. $\square$

## Appendix B: A Note on the Numerical Algorithm Adopted

The systems considered consist of mixed Ordinary–Partial Differential Equations. All differential equations are first order, non linear and leave $\mathopen [0, +\infty \mathclose [$ invariant. The particular structure of the convective parts in the PDEs, where both independent variables are times, suggests to use a simple upwind scheme (LeVeque 2002, Sect. 4.2), using the same mesh $\Delta t$ for all independent variables (*t* and $\tau$), although they vary in different time interval. The right hand sides of all equations are computed through a first order forward Euler method, taking care that equality (2.6) [or its analog (4.4)] keeps holding at each time step.

To prevent the *S* variable getting negative when it is near to 0, we employed a simple predictor-corrector method. Whenever $S (t+\Delta t)$ gets negative, the value of *p*(*t*) is recomputed, consistently with the equation, so that $S (t+\Delta t) =0$.
